# The Effects of Glucagon-Like Peptide-1 Receptor Agonists and Dipeptydilpeptidase-4 Inhibitors on Blood Pressure and Cardiovascular Complications in Diabetes

**DOI:** 10.1155/2021/6518221

**Published:** 2021-06-30

**Authors:** Habib Yaribeygi, Farin Rashid Farrokhi, Mohammed Altigani Abdalla, Thozhukat Sathyapalan, Maciej Banach, Tannaz Jamialahmadi, Amirhossein Sahebkar

**Affiliations:** ^1^Research Center of Physiology, Semnan University of Medical Sciences, Semnan, Iran; ^2^Chronic Kidney Disease Research Center, Shahid Beheshti University of Medical Sciences, Tehran, Iran; ^3^Academic Diabetes, Endocrinology and Metabolism, Hull York Medical School, University of Hull, UK; ^4^Department of Hypertension, WAM University Hospital in Lodz, Medical University of Lodz, Zeromskiego 113, Lodz, Poland; ^5^Department of Food Science and Technology, Quchan Branch, Islamic Azad University, Quchan, Iran; ^6^Department of Nutrition, Faculty of Medicine, Mashhad University of Medical Sciences, Mashhad, Iran; ^7^Biotechnology Research Center, Pharmaceutical Technology Institute, Mashhad University of Medical Sciences, Mashhad, Iran; ^8^Applied Biomedical Research Center, Mashhad University of Medical Sciences, Mashhad, Iran; ^9^School of Pharmacy, Mashhad University of Medical Sciences, Mashhad, Iran

## Abstract

Glucagon-like peptide-1 receptor (GLP-1R) agonists are a class of newly introduced antidiabetic medications that potentially lower blood glucose by several molecular pathways. DPP-4 inhibitors are the other type of novel antidiabetic medications which act by preventing GLP-1 inactivation and thereby increasing the activity levels of GLP-1, leading to more glucose-induced insulin release from islet *β*-cells and suppression of glucagon release. Most patients with diabetes have concurrent hypertension and cardiovascular disorder. If antihyperglycemic agents can attenuate the risk of hypertension and cardiovascular disease, they will amplify their overall beneficial effects. There is conflicting evidence on the cardiovascular benefits of GLP-1R induction in laboratory studies and clinical trials. In this study, we have reviewed the main molecular mechanisms by which GLP-1R induction may modulate the cardiovascular function and the results of cardiovascular outcome clinical trials.

## 1. Introduction

The global incidence of diabetes mellitus is growing rapidly [1]. This chronic disorder is accompanied by metabolic derangements and activation of various pathophysiologic pathways leading to tissue dysfunction [2]. Nowadays, diabetes complications are a leading cause of disability and mortality, especially in the elderly worldwide [3]. Hence, various therapeutic guidelines and antidiabetic agents have been developed for normalising blood glucose and preventing diabetes-related complications [4, 5]. Diabetes complications are classified mainly as microvascular and macrovascular complications, both of which are worsened by hemodynamic variations and increased blood pressure (BP) [6, 7].

Moreover, it is well established that hypertension coexists in a significant proportion of patients with diabetes [8, 9]. Hence, if an antidiabetic medication modulates hemodynamic changes and normalises hypertension, it can be more beneficial against diabetes-related complications [10]. While we have some evidence about the effects of classic antidiabetic agents on hemodynamic variations [11–13], there is not much literature about antihyperglycemic medications. Therefore, in this current study, we present the latest evidence about glucagon-like peptide-1 receptor agonists (GLP-1RA) and dipeptydilpeptidase-4 inhibitors (DPP-4i), which are a relatively newer class of antihyperglycemic agents on hypertension in the diabetic milieu.

## 2. GLP-1RA and DPP-4i

GLP-1RA is a class of newly introduced antidiabetic medications that are FDA approved in 2010 to manage patients with diabetes [9]. They act as an agonist to GLP-1 receptors and mimic the effects of incretin hormones [14, 15]. Incretin is a family of metabolic hormones that includes intestinal GLP-1 and gastric inhibitory peptide (GIP) and reduces postprandial blood glucose by inhibiting glucagon secretion from pancreatic *α*-cells and stimulating insulin release from *β*-cell in a blood glucose-dependent manner [14, 16, 17]. Moreover, they can provide additional effects such as delayed gastric emptying, suppression of appetite, declining nutrient absorption in the gut, improvement of lipid metabolism, and inhibition of pancreatic *β*-cell apoptosis [16, 18–25]. These antihyperglycemic agents activate their specific receptor known as GLP-1R, predominantly located in pancreatic *β*-cell [17]. GLP-1R is a member of G protein-coupled receptors. Its activation is followed by higher production of cAMP (cyclic adenosine monophosphate), cellular depolarisation, and augmentation in intracellular calcium concentration insulin secretion from pancreatic *β*-cells [17, 26].

DPP-4 inhibitors are the other type of novel antidiabetic medications which act by preventing GLP-1 inactivation and thereby increasing the activity levels of GLP-1, leading to more glucose-induced insulin release from islet *β*-cells and suppression of glucagon release [27, 28]. After posttranslational processing of preglucagon (PG) peptides in intestinal L cells, at least four separate forms of PG were secreted, all of which can be inactivated by the dipeptidyl peptidase-4 (DPP-4) enzyme by removing the two amino acids from the N-terminal residue [29]. Therefore, the DPP-4 inhibitors have the same antihyperglycemic effects as GLP-1 agonists, although they have some differences in body weight and risk of adverse effects [27].

## 3. Classification of Diabetes Mellitus

The two major diabetes mellitus (DM) are type 1 and type 2 diabetes [30]. Type 1 DM (T1DM) accounts for about 5-10% of all patients with diabetes and results from autoimmune destruction of beta-cells of the pancreas and absolute deficiency of insulin [30]. Type 2 DM (T2DM) (NIDDM) is the most prevalent form of DM, which accounts for about 90-95% of diabetic subjects and is mainly associated with various pathologies, including insulin resistance and beta-cell dysfunction [30]. Gestational diabetes is another type of DM that happens in pregnant women mainly via hormonal variation-induced insulin resistance in pregnancy [31]. Other forms of DM are maturity-onset diabetes of young with autosomal dominant inheritance, LADA (Latent Autoimmune Diabetes in Adults), which is primarily considered one subclass of TIDM and secondary diabetes due to other pathologies such as chronic pancreatitis and secondary to medications such as steroids. [32].

## 4. Diabetes and Vascular Disease

Alteration in vascular homeostasis due to smooth muscle and endothelial cell dysfunction is the main cause of vascular disease associated with diabetes. Both macro- and microvascular diabetes complications are primarily due to prolonged exposure to high glucose level which also clusters other problems such as hypertension [33]. Initially, hyperglycemia leads to an imbalance between nitric oxide (NO) produced by the endothelial cell and the reactive oxygen species (ROS). NO is an indicative of vascular health and causes vasodilation by its effect on the vascular smooth muscle cells. The reduction in endothelial-derived NO increases the proinflammatory cytokines resulting in endothelial dysfunction. Moreover, hyperglycemia also increases the production of advanced glycation end products (AGEs) which in turn deactivate NO and induce vascular dysfunction [34].

## 5. Hypertension as a Main Upstream Event in Diabetes Complications

Many forms of diabetes complications such as diabetic nephropathy, diabetic retinopathy, stroke, and DM-related cardiovascular disorders such as atherosclerosis are closely associated with hemodynamic variations and hypertension [35]. Emerging studies have well demonstrated that the simultaneous presence of hypertension is a main upstream event and potent risk factor which can induce or exacerbate the progression of diabetes complications [35, 36]. For example, Grzeszczak et al. reported that extensive uncontrolled hemodynamic aberrations accompany diabetic nephropathy due to the morphological and functional alteration in the kidneys and systemic and intraglomerular hypertension in patients with diabetes [37]. However, glomerular hyperfiltration which is considered a hemodynamic abnormality in kidneys also acts as an independent risk factor for diabetic nephropathy in patients with diabetes [38, 39].

Moreover, hypertension (HTN) in patients with diabetes is associated with cardiovascular complications and atherosclerosis, which markedly increase the risk of stroke and myocardial infarction [40–42]. Likewise, other prevalent forms of diabetes complications such as diabetic neuropathy and diabetic retinopathy are potentially influenced by molecular pathways induced by hemodynamic changes and systemic hypertension [10, 35, 43, 44]. Therefore, the management of hypertension in patients with diabetes is of crucial importance for researchers and physicians [10].

## 6. The Molecular Mechanism and Signalling Pathway by Which GLP-1RAs and DPP-4is Exert Their Effects

There is significant evidence that GLP-1RAs and DPP-4i enhance the insulin activity and the glucose uptake in animal and human muscle [45]. It has been proposed that GLP-1 enhances glucose disposal in an insulin-independent mechanism [46]. The GLP-1 receptors are expressed in the brain and *β*-cells of the pancreas where GLP-1 exerts multiple actions. In the pancreas, it stimulates insulin secretion by many molecular pathways including the release of cyclic adenosine monophosphate (cAMP) by activating *β*-arrestin-1 (*β*ARR1), activates the voltage Ca^2+^ channels, and induces the Ca^2+^ influx which raises the intracellular Ca^2+^ and stimulates the insulin release [47, 48]. GLP-1 also stimulates *β*-cell proliferation by downregulating PI3-K, mitogen-activated protein kinase (MAPK), and p38, [49–51]. There is evidence suggesting that GLP-1 possesses anti-inflammatory properties. It suppresses inflammation by reducing secretion of inflammatory cytokines such as interleukin-1 *β* (IL-1*β*), tumour necrosis factor-*β* (TNF-*β*), and interleukin (IL) [52]. Moreover, GLP-1RAs reduce the stress in the endoplasmic reticulum (ER) by modulating the protein kinase R-like endoplasmic reticulum (PERK) pathway and activate the transcription factor 4 (ATF4) and CHOP (C/EBP homologous protein) [53]. There is growing evidence that GLP-1 A reverses the vascular remodelling by downregulating the matrix metalloproteinase 1 (MMP1), extracellular-regulated protein kinase 1/2 (ERK1/2), and nuclear factor kappa-*β* (NF-K*β*) [54]. Therefore, via this mechanism, GLP-1RAs are reducing cardiac and vascular inflammation.

## 7. Possible Links between GLP-1RA and DDP-4i and Blood Pressure

Some evidence suggested that GLP-1 agonists and DPP-4i can influence the hemodynamic state and modify BP [55–57]. We have reviewed all possible mechanisms associated with this class of agents and hemodynamics in the following sections (Table 1). Since these two antidiabetic agents have the same basis of molecular effects, we have reviewed them together.

## 8. GLP-1RA and DPP-4i and Vascular Endothelial Function

Vascular endothelial cells have a significant role in the homeostasis of cardiovascular function and BP homeostasis [58, 59]. GLP-1 agonists may improve vascular endothelial function in the diabetic milieu [58, 60, 61]. In a study by Basu et al., it was found that GLP-1 stimulated acetylcholine-induced vasodilatation, improved vascular relaxation, reduced diastolic BP, and has direct beneficial effects on endothelial function in patients with T1DM [61]. Similarly, Liu et al. reported that GLP-1 agonists improve endothelial cell function and regulate vascular contractions by promoting nitric oxide (NO) release and suppressing oxidative stress [58]. On the other hand, Ceriello et al. demonstrated that GLP-1 agonists attenuated endothelial dysfunction by inhibiting oxidative stress and inflammation in patients with T1DM [59]. Also, Liu and coworkers demonstrated that DPP-4i reduced BP by improving endothelial function in hypertensive rats [62]. So improvement of endothelial function can be a way for GLP-1 agonists to normalise BP [61].

However, not all studies showed beneficial effects [63]. For example, Widlansky et al. demonstrated that DPP-4i has no significant acute effect on endothelial dysfunction in patients with T2DM [63]. While Romacho et al. reported that DPP-4i improves endothelial function by activation of PAR2 (protease-activated receptor 2) and release of thromboxane-A2 in mice [64], Nomoto et al. provided evidence indicating that DPP-4i does not improve endothelial dysfunction [65], suggesting that more evidence is needed to show beneficial effects of these agents on endothelial function.

## 9. GLP-1RA and DPP-4i and Heart Rate

There is growing evidence that GLP-1 receptor agonists may increase the heart rate (HR) [66–69]; however, some studies suggest no significant effects [61, 63]. On the other hand, most of this evidence relied on the positive chronotropic effect of these drugs on HR and suggested that this may be due to modulating the autonomic nervous system, leading to more sympathetic activity [61, 63, 66, 70].

## 10. GLP-1RA and DPP-4i and Oxidative Stress

Oxidative stress due to free radical overload is a main upstream event in many pathologic conditions, including (HTN) [71–73]. Some evidence indicated that GLP-1 receptor activation might improve oxidative stress [74]. This could be mediated via inhibition of free radical generation by cyclooxygenase 2 and/or NADPH oxidase downregulation of the MAPK (mitogen-activated protein kinase) pathway [58, 59, 75]. Also, they may protect against oxidative damage in vascular cells via inhibition of PKC-*α* (protein kinase c-*α*) and NF-*κ*b (nuclear factor kappa b) signalling and activation of the Nrf2 nuclear factor and upregulation of protective antioxidative enzymes such as SOD (superoxide dismutase) and CAT (catalase) [76].

However, there is only minimal direct evidence about the effects of GLP-1 on oxidative stress-induced HTN [77]. Koren and coworkers demonstrated that sitagliptin ameliorated oxidative stress in vascular cells without remarkable effects on BP [77]. Also, Alam et al. demonstrated that sitagliptin inhibits oxidative stress in vascular cells and improves arterial function of the kidneys and heart of rats [78]. Further evidence is still required to elucidate the exact role of GLP-1 induction in oxidative stress-dependent hypertension.

## 11. GLP-1RA and DPP-4i and Nitric Oxide

Nitric oxide (NO) plays a significant role in vascular homeostasis and the normal physiologic function of the cardiovascular system [79]. Liu et al. demonstrated that GLP-1 receptor activation protects endothelial cells by upregulating NO synthesis [58]. Ding and Zhang showed that the GLP-1 agonist induced NO mRNA expression and improved NO synthesis in endothelial cells of the umbilical vein [80]. Also, Chai et al. found that GLP-1 receptor activity is associated with endothelial NO synthesis and improvement in microvascular blood flow [81]. Moreover, Dong and colleagues reported that GLP-1 acutely stimulated eNOS phosphorylation at Ser^1177^ and NO production by a PKA-dependent pathway, leading to improved microvascular muscle blood flow [82].

We have similar evidence concerning DPP-4i and NO synthesis [83]. Mason et al. illustrated that DPP-4 inhibition with saxagliptin reduced BP by enhancing NO levels in hypertensive rats [83]. Also, Al-Awar and coworkers reported that sitagliptin, one of the DPP-4is, improved vascular function and reduced BP by NO-dependent molecular mechanisms in rats [84]. So we suggest that GLP-1 induction by either agonists or DPP-4i results in more NO synthesis leading to better vascular smooth muscle cell function and lower levels of BP [82, 84].

## 12. GLP-1RA and DPP-4i and Central Nervous System

Central nervous system (CNS) activity, especially the autonomic nervous system (ANS), has a prominent role in cardiovascular function and control of BP [67]. Emerging evidence strongly suggests that GLP-1 receptor activation increases sympathetic activity and HR elevation leading to hypertension [66, 67]. GLP-1R expressed in many regions of CNS is involved in the lateral septum, the posterodorsal tegmental nucleus, the thalamus and hypothalamus, the subcortical organ, the area postrema, the interpeduncular nucleus, the nucleus of the solitary tract, and the inferior olive, and so, its activity makes potent inotropic effects leading to more HR and BP [85, 86].

Andrews et al. demonstrated that the GLP-1 agonist of exendin-4 induced sympathetic activity and suppressed vagal nerves in human [87]. Also, Nakatani and coworkers reported that GLP-1R stimulation by liraglutide induces sympathetic activity and increases HR and BP in patients with T2DM [66]. Marney et al. provided the same evidence about DPP-4i, indicating that they intensify sympathetic activity in human [88]. So it is strongly suggested that GLP-1R induction increases ANS activity and elevates BP [66, 88].

## 13. GLP-1RA and DPP-4i and Renal Function

A healthy renal system is required for the normal function of the cardiovascular system and maintaining physiologic BP [89]. Renal dysfunctions accompany many cases of HTN, and improvement in renal function improves the cardiovascular outcome [89]. Recent evidence suggests that GLP-1R activation may modulate water and electrolyte homeostasis and improve renal microvascular function in the diabetic milieu [90]. Marney and colleagues demonstrated that sitagliptin improved renal blood flow in the diabetic milieu [88]. Boye et al. reported that GLP-1R induction was accompanied by a slower deterioration in eGFR (estimated glomerular filtration rate) than that of other antihyperglycemic agents [91].

Dieter et al. provided strong evidence on GLP-1R stimulation improving renal function by mechanisms beyond controlling hyperglycemia such as anti-inflammation and natriuresis in patients with diabetes [92]. Liu et al. showed that exenatide decreased BP by improving renal impairment in hypertensive rats [62]. Also, Hirata and Kume found that exendin-4 improved renal sufficiency and ameliorated HTN in angiotensin-induced hypertensive rats [93]. Moreover, Liu and coworkers demonstrated that the GLP-1 agonist reduced BP by improving renal function in hypertensive rats [94]. Said and coworkers established that DPP-4i with alogliptin reduced BP by improving renal sufficiency in patients with T2DM [95]. Alter et al. demonstrated that DPP-4 inhibition in hypertensive rats normalised BP by improving renal function [96]. This evidence strongly suggests that GLP-1R induction can improve BP control at least partly via improvement in renal sufficiency [94, 96].

## 14. GLP-1RA and DPP-4i and Pulmonary Artery Pressure

Recent evidence suggests that GLP-1 receptor activation could potentially reduce pulmonary artery pressure (PAP) in patients with diabetes [97]. Lee and colleagues found that liraglutide reduces PAP via eNOS/sGC/PKG and rho kinase pathways in diabetic rats (soluble guanylyl cyclase (sGC), protein kinase G (PKG)) [97]. Woo et al. demonstrated that exenatide diminished pulmonary capillary wedge pressure and improved coronary blood flow in patients with T2DM who have cardiovascular disorders [98]. Pirozzi and Diaz presented a case report implying that DPP-4 inhibition with vildagliptin reduces PAP by NO synthesis induction and vascular potassium channel activation [99].

Honda and coworkers showed that the GLP-1R agonist prevents hypoxia-dependent HTN in pulmonary vessels of mice [100]. Further evidence has been provided by Hosokawa and coworkers in 2014, where they indicated that incretin drugs of GLP-1 agonists or DPP-4i could potentially modulate PAP in the hypertensive milieu [101]. Hence, part of the beneficial cardiovascular effects of these agents could potentially be mediated by modulating the pulmonary arterial pressure [101].

## 15. GLP-1RA and DPP-4i and Renin-Angiotensin System (RAS)

It has been shown that GLP-1R activation can potentially interact with RAS activity [102]. Skov et al. provided the first evidence showing that GLP-1 infusion declined angiotensin II and induced natriuresis in healthy young men [102]. Le et al. demonstrated that GLP-1R activation by exendin-4 declined intrarenal RAS activity, leading to a lower Ang II-mediated TGF-*β*1/Smad3 signalling pathway in mice (transforming growth factor-beta (TGF-*β*1), SMAD family member 3 (Smad3)) [103].

However, recent studies did not show similar effects by DPP-4 inhibitors [104]. Hubers et al. reported that DPP-4 inhibition could induce vasoconstriction in patients with T2DM [104]. Also, Cooper et al. suggested that concomitant use of ACE (angiotensin-converting enzyme) inhibitors and DPP-4i potentiates their effect on RAS activity [105]. More studies are needed to elucidate the possible relationships between RAS activity and GLP-1R agonists and DPP-4i.

## 16. Other Possible Effects

**Figure 1:**
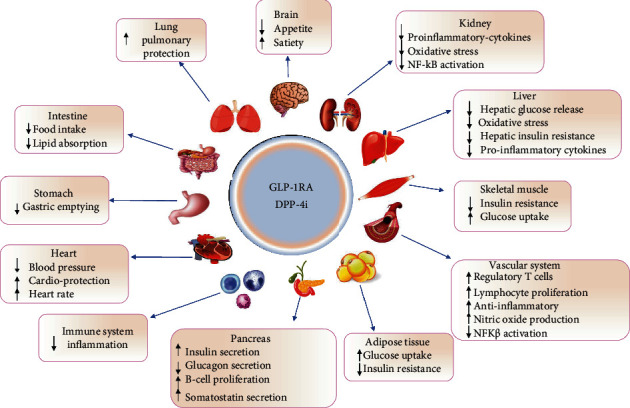
The mechanism by which GLP-1RA and DPP-4i act on various organs.

In addition to the pathways mentioned above, the other potential mechanisms suggested are ANP release induction [106], natriuresis induction [90, 92, 102, 107], anti-inflammatory effects [92], improvement in lipid metabolism, and lower risk for atherosclerosis [108]. The exact roles of these agents on the cardiovascular system and BP need to be elucidated in further studies (Figure 1).

## 17. GLP-1RA and DPP-4i and Cardiovascular Disease in Clinical Trials

This section reviewed the clinical studies on the effects of the GLP-1R agonist on cardiovascular diseases in healthy individuals and patients with diabetes (Table 2).

## 18. Conclusion

GLP-1R induction provides potent antihyperglycemic effects. However, there is conflicting evidence on their possible roles in cardiovascular disorders. It improves endothelial cell function and regulates vascular contractions by promoting nitric oxide release and suppressing oxidative stress. Both GLP-1 and DPP4is have significant effects on the autonomic nervous system by increasing sympathetic activity. Moreover, they also improve renal function in patients with diabetes by regulating electrolytes. The cumulative evidence from the recent cardiovascular outcome trials suggests that the effects of GLP-1R activation have a beneficial effect on blood pressure and cardiovascular diseases. However, a robust meta-analysis is needed to compare the controversial results of the different papers in the current literature.

## Figures and Tables

**Table 1:** Mechanisms by which GLP-1 modulates the cardiovascular system and blood pressure.

Mechanisms	Effects of GLP-1R and DPP-4i on the cardiovascular system and blood pressure	Ref.
Vascular endothelial function	Improves function of vascular endothelial cells	[58, 60–62]
Heart rate	Increases heart rate leading to higher blood pressure	[66–69]
Oxidative stress	Improves redox state leading to the promoted function of the cardiovascular system; little is known about the direct effect on the cardiovascular system and blood pressure	[58, 59, 74–78]
Nitric oxide	Induces nitric oxide production in vascular endothelial cells	[80–84]
Central nervous system	Activates the sympathetic nervous system leading to a higher heart rate and blood pressure	[66, 85, 86, 88]
Renal function	Indirectly improves cardiovascular function by promoting renal sufficiency	[90–96]
Pulmonary artery pressure	Declines pulmonary artery pressure	[97–101]
Renin-angiotensin system	Modulates the renin-angiotensin system, needs more investigations yet	[86, 104]

**Table 2:** Significant clinical findings on the effects of GLP-1R activation on the cardiovascular system and blood pressure.

Trial number	Population of study	Used drug(s)	Effects on vascular function and BP	Ref.
—	30 patients with T1DM	GLP-1 infusion	Improves endothelial function, no significant effects on BP	[59]
NCT01859793	38 patients with T2DM	Sitagliptin	No significant effects on BP	[63]
UMIN000017770	60 patients with T2DM	Liraglutide and lixisenatide	Increase in HR and BP	[66]
NIHMS360699	24 patients with metabolic syndrome	Sitagliptin	Induces ANS (sympathetic) activity and increases BP	[88]
—	76 patients with T2DM	Alogliptin	Reduced BP by improving renal sufficiency	[95]
NCT01580514	58 patients with T2DM and cardiac dysfunction	Exenatide	Diminishes pulmonary capillary wedge pressure and improved coronary blood flow	[98]
NTC00294723	746 patients with early T2DM	Liraglutide	Potentially reduces blood pressure after 52 weeks	[109]
NCT00331851	581 patients with T2DM	Liraglutide	Has no significant effects on systolic blood pressure	[110]
—	929 patients with T2DM	Liraglutide	Reduced systolic blood pressure	[111]
NCT01333163	12 healthy young men	GLP-1 infusion	Declined angiotensin II and induces natriuresis	[102]
NCT01179048	9340 patients with T2DM	Liraglutide	Reduces the risk of cardiovascular disorders	[112]
NCT02465515	9463 patients with T2DM	Albiglutide	Diminishes the risk of diabetes-induced cardiovascular complications	[113]
NCT01147250	6068 patients with T2DM	Lixisenatide	No significant effects on diabetes-induced cardiovascular diseases	[114]
NCT01144338	14752 patients with T2DM	Exenatide	No significant effects on diabetes-induced cardiovascular diseases	[115]
NCT01755572	22 hypertensive subjects with T2DM	Liraglutide	While inducing natriuresis, it had no significant effects on blood pressure	[99]
NCT01664676	11 male patients with T2DM	Liraglutide	Reduces blood pressure in the short term by suppressing RAS activity and renal protection	[101]
—	11 subjects with T2DM	Liraglutide	Was unable to correct the abnormal elevation in nocturnal BP known to occur in patients with T2DM	[104]
NCT01720446	3297 patients with T2DM	Semaglutide	Reduces the risk of diabetes-induced cardiovascular problems	[116]

## References

[1] Mayer-Davis E. J., Lawrence J. M., Dabelea D. (2017). Incidence trends of type 1 and type 2 diabetes among youths, 2002–2012. *New England Journal of Medicine*.

[2] Hirsch I. B. (2015). Glycemic variability and diabetes complications: does it matter? Of course it does!. *Diabetes Care*.

[3] Gregg E. W., Sattar N., Ali M. K. (2016). The changing face of diabetes complications. *The lancet Diabetes & endocrinology*.

[4] Bergenstal R. M. (2015). Glycemic variability and diabetes complications: does it matter? Simply put, there are better glycemic markers!. *Diabetes Care*.

[5] Kuniss N., Freyer M., Müller N., Kielstein V., Müller U. A. (2019). Expectations and fear of diabetes-related long-term complications in people with type 2 diabetes at primary care level. *Acta diabetologica*.

[6] Domingueti C. P., Dusse L. M. S. A., Carvalho M. G., de Sousa L. P., Gomes K. B., Fernandes A. P. (2016). Diabetes mellitus: the linkage between oxidative stress, inflammation, hypercoagulability and vascular complications. *Journal of Diabetes and its Complications*.

[7] Lozano I., van der Werf R., Bietiger W. (2016). High-fructose and hih-fat diet-induced disorders in rats: impact on diabetes risk, hepatic and vascular complications. *Nutrition & metabolism*.

[8] Raina S. K., Chander V., Raina S., Kumar D., Grover A., Bhardwaj A. (2015). Hypertension and diabetes as risk factors for dementia: a secondary post-hoc analysis from north-west India. *Annals of Indian Academy of Neurology*.

[9] Yaribeygi H., Atkin S. L., Pirro M., Sahebkar A. (2018). A review of the anti-inflammatory properties of antidiabetic agents providing protective effects against vascular complications in diabetes. *Journal of cellular physiology*.

[10] de Boer I. H., Bangalore S., Benetos A. (2017). Diabetes and hypertension: a position statement by the American Diabetes Association. *Diabetes Care*.

[11] Dean A., Nilsen M., Loughlin L., Salt I. P., MacLean M. R. (2016). Metformin reverses development of pulmonary hypertension via aromatase inhibition. *Hypertension*.

[12] Cosenso-Martin L. N., Giollo-Júnior L. T., Fernandes L. A. B. (2018). Effect of vildagliptin versus glibenclamide on endothelial function and arterial stiffness in patients with type 2 diabetes and hypertension: a randomized controlled trial. *Acta diabetologica*.

[13] Brands M. W. (2018). Role of insulin-mediated antinatriuresis in sodium homeostasis and hypertension. *Hypertension*.

[14] Drucker D. J., Nauck M. A. (2006). The incretin system: glucagon-like peptide-1 receptor agonists and dipeptidyl peptidase-4 inhibitors in type 2 diabetes. *The Lancet*.

[15] Islam M. (2016). Insulinotropic effect of herbal drugs for management of diabetes mellitus: a congregational approach. *Biosensors Journal*.

[16] Meier J. J. (2012). GLP-1 receptor agonists for individualized treatment of type 2 diabetes mellitus. *Nature Reviews Endocrinology*.

[17] Baggio L. L., Drucker D. J. (2007). Biology of incretins: GLP-1 and GIP. *Gastroenterology*.

[18] Yaribeygi H., Maleki M., Sathyapalan T., Jamialahmadi T., Sahebkar A. (2021). Antioxidative potentials of incretin-based medications: a review of molecular mechanisms. *Oxidative Medicine and Cellular Longevity*.

[19] Ranjbar G., Mikhailidis D. P., Sahebkar A. (2019). Effects of newer antidiabetic drugs on nonalcoholic fatty liver and steatohepatitis: Think out of the box!. *Metabolism: Clinical and Experimental*.

[20] Yaribeygi H., Maleki M., Sathyapalan T., Jamialahmadi T., Sahebkar A. (2020). Incretin-based therapies and renin-angiotensin system: Looking for new therapeutic potentials in the diabetic milieu. *Life Sciences*.

[21] Yaribeygi H., Ashrafizadeh M., Henney N. C., Sathyapalan T., Jamialahmadi T., Sahebkar A. (2020). Neuromodulatory effects of anti-diabetes medications: A mechanistic review. *Pharmacological Research*.

[22] Yaribeygi H., Maleki M., Sathyapalan T., Jamialahmadi T., Sahebkar A. (2020). Anti-inflammatory potentials of incretin-based therapies used in the management of diabetes. *Life Sciences*.

[23] Yaribeygi H., Atkin S. L., Jamialahmadi T., Sahebkar A. (2007). A review on the effects of new anti-diabetic drugs on platelet function. *Endocrine, Metabolic and Immune Disorders - Drug Targets*.

[24] Scott K. A., Moran T. H. (2007). The GLP-1 agonist exendin-4 reduces food intake in nonhuman primates through changes in meal size. *American Journal of Physiology-Regulatory, Integrative and Comparative Physiology*.

[25] Ding X., Saxena N. K., Lin S., Gupta N., Anania F. A. (2006). Exendin-4, a glucagon-like protein-1 (GLP-1) receptor agonist, reverses hepatic steatosis in Ob/Ob mice. *Hepatology*.

[26] Wootten D., Simms J., Koole C. (2011). Modulation of the glucagon-like peptide-1 receptor signaling by naturally occurring and synthetic flavonoids. *Journal of Pharmacology and Experimental Therapeutics*.

[27] American Diabetes Association (2018). 2. Classification and diagnosis of diabetes: *standards of medical care in diabetes—2018*. *Diabetes Care*.

[28] Ahren B. (2007). DPP-4 inhibitors. *Best Practice & Research Clinical Endocrinology & Metabolism*.

[29] Brubaker P. L. (2006). The glucagon-like peptides: pleiotropic regulators of nutrient homeostasis. *Annals of the New York Academy of Sciences*.

[30] Association AD (2014). Diagnosis and classification of diabetes mellitus. *Diabetes care*.

[31] de Faria M. J. (2013). Classification of Diabetes. *Diabetes*.

[32] O’Neal K. S., Johnson J. L., Panak R. L. (2016). Recognizing and appropriately treating latent autoimmune diabetes in adults. *Diabetes Spectrum*.

[33] Creager M. A., Lüscher T. F., Cosentino F., Beckman J. A. (2003). Diabetes and vascular disease: pathophysiology, clinical consequences, and medical therapy: part I. *Circulation*.

[34] Paneni F., Beckman J. A., Creager M. A., Cosentino F. (2013). Diabetes and vascular disease: pathophysiology, clinical consequences, and medical therapy: part I. *European Heart Journal*.

[35] Yamazaki D., Hitomi H., Nishiyama A. (2018). Hypertension with diabetes mellitus complications. *Hypertension Research*.

[36] Long A. N., Dagogo-Jack S. (2011). Comorbidities of diabetes and hypertension: mechanisms and approach to target organ protection. *The Journal of Clinical Hypertension*.

[37] Grzeszczak W., Wystrychowski G., Franek E. (2013). The role of haemodynamic and metabolic factors in the development of diabetic nephropathy. *Postępy Nauk Medycznych*.

[38] Tonneijck L., Muskiet M., Smits M. M. (2017). Glomerular hyperfiltration in diabetes: mechanisms, clinical significance, and treatment. *Journal of the American Society of Nephrology: JASN*.

[39] Tuttle K. R. (2017). Back to the future: glomerular hyperfiltration and the diabetic kidney. *Diabetes*.

[40] Howard B. V., Roman M. J., Devereux R. B. (2008). Effect of lower targets for blood pressure and LDL cholesterol on atherosclerosis in diabetes: the SANDS randomized trial. *Journal of the American Medical Association*.

[41] Turnbull F., Neal B., Algert C. (2005). Effects of different blood pressure-lowering regimens on major cardiovascular events in individuals with and without diabetes mellitus: results of prospectively designed overviews of randomized trials. *Archives of Internal Medicine*.

[42] Prenner S. B., Chirinos J. A. (2015). Arterial stiffness in diabetes mellitus. *Atherosclerosis*.

[43] Smith D. I., Tran H. T., Poku J. (2018). Hemodynamic considerations in the pathophysiology of peripheral neuropathy. Blood pressure-from bench to bed.

[44] Bernabeu M. O., Lu Y., Lammer J., Aiello L. P., Coveney P. V., Sun J. K. Characterization of parafoveal hemodynamics associated with diabetic retinopathy with adaptive optics scanning laser ophthalmoscopy and computational fluid dynamics.

[45] McClenaghan N. H. (2007). Physiological regulation of the pancreatic {beta}-cell: functional insights for understanding and therapy of diabetes. *Experimental Physiology*.

[46] Drucker D. J. (2001). Minireview: the glucagon-like peptides. *Endocrinology*.

[47] Meloni A. R., DeYoung M. B., Lowe C., Parkes D. G. (2013). GLP-1 receptor activated insulin secretion from pancreatic *β*‐cells: mechanism and glucose dependence. *Diabetes, Obesity & Metabolism*.

[48] Papaetis G. S. (2014). Incretin-based therapies in prediabetes: current evidence and future perspectives. *World Journal of Diabetes*.

[49] Buteau J., Foisy S., Rhodes C. J., Carpenter L., Biden T. J., Prentki M. (2001). Protein kinase Czeta activation mediates glucagon-like peptide-1-induced pancreatic beta-cell proliferation. *Diabetes*.

[50] Buteau J., Foisy S., Joly E., Prentki M. (2003). Glucagon-like peptide 1 induces pancreatic beta-cell proliferation via transactivation of the epidermal growth factor receptor. *Diabetes*.

[51] Matarese A., Gambardella J., Lombardi A., Wang X., Santulli G. (2020). miR-7 regulates GLP-1-mediated insulin release by targeting *β*-Arrestin 1. *Cells*.

[52] Guo C., Huang T., Chen A. (2016). Glucagon-like peptide 1 improves insulin resistance *in vitro* through anti-inflammation of macrophages. *Brazilian Journal of Medical and Biological Research*.

[53] Jiang Y., Wang Z., Ma B. (2018). GLP-1 improves adipocyte insulin sensitivity following induction of endoplasmic reticulum stress. *Frontiers in Pharmacology*.

[54] Fan S. H., Xiong Q. F., Wang L., Zhang L. H., Shi Y. W. (2020). Glucagon-like peptide 1 treatment reverses vascular remodelling by downregulating matrix metalloproteinase 1 expression through inhibition of the ERK1/2/NF-*κ*B signalling pathway. *Molecular and Cellular Endocrinology*.

[55] Sun F., Wu S., Guo S. (2015). Impact of GLP-1 receptor agonists on blood pressure, heart rate and hypertension among patients with type 2 diabetes: a systematic review and network meta-analysis. *Diabetes research and clinical practice*.

[56] Wang B., Zhong J., Lin H. (2013). Blood pressure-lowering effects of GLP-1 receptor agonists exenatide and liraglutide: a meta-analysis of clinical trials. *Diabetes, Obesity and Metabolism*.

[57] Liakos C. I., Papadopoulos D. P., Sanidas E. A. (2021). Blood pressure-lowering effect of newer antihyperglycemic agents (SGLT-2 inhibitors, GLP-1 receptor agonists, and DPP-4 inhibitors). *American Journal of Cardiovascular Drugs*.

[58] Liu L., Liu J., Huang Y. (2015). Protective effects of glucagon-like peptide 1 on endothelial function in hypertension. *Journal of cardiovascular pharmacology*.

[59] Ceriello A., Novials A., Ortega E. (2013). Glucagon-like peptide 1 reduces endothelial dysfunction, inflammation, and oxidative stress induced by both hyperglycemia and hypoglycemia in type 1 diabetes. *Diabetes Care*.

[60] Lovshin J., Cherney D. (2015). GLP-1R agonists and endothelial dysfunction: more than just glucose lowering?. *Diabetes*.

[61] Basu A., Charkoudian N., Schrage W., Rizza R. A., Basu R., Joyner M. J. (2007). Beneficial effects of GLP-1 on endothelial function in humans: dampening by glyburide but not by glimepiride. *American Journal of Physiology-Endocrinology and Metabolism*.

[62] Liu L., Liu J., Wong W. T. (2012). Dipeptidyl peptidase 4 inhibitor sitagliptin protects endothelial function in hypertension through a glucagon–like peptide 1–dependent mechanism. *Hypertension*.

[63] Widlansky M. E., Puppala V. K., Suboc T. M. (2017). Impact of DPP-4 inhibition on acute and chronic endothelial function in humans with type 2 diabetes on background metformin therapy. *Vascular Medicine*.

[64] Romacho T., Vallejo S., Villalobos L. A. (2016). Soluble dipeptidyl peptidase-4 induces microvascular endothelial dysfunction through proteinase-activated receptor-2 and thromboxane A2 release. *Journal of hypertension*.

[65] Nomoto H., Miyoshi H., Nakamura A. (2017). Do DPP-4 inhibitors improve endothelial cell function?. *Current Trends in Cardiology*.

[66] Nakatani Y., Kawabe A., Matsumura M. (2016). Effects of GLP-1 receptor agonists on heart rate and the autonomic nervous system using Holter electrocardiography and power spectrum analysis of heart rate variability. *Diabetes Care*.

[67] Gardiner S. M., March J. E., Kemp P. A., Bennett T. (2006). Mesenteric vasoconstriction and hindquarters vasodilatation accompany the pressor actions of exendin-4 in conscious rats. *Journal of Pharmacology and Experimental Therapeutics*.

[68] Barragan J. M., Rodríguez R. E., Eng J., Blázquez E. (1996). Interactions of exendin-(9–39) with the effects of glucagon-like peptide-1-(7–36) amide and of exendin-4 on arterial blood pressure and heart rate in rats. *Regulatory peptides*.

[69] Yamamoto H., Lee C. E., Marcus J. N. (2002). Glucagon-like peptide-1 receptor stimulation increases blood pressure and heart rate and activates autonomic regulatory neurons. *The Journal of clinical investigation*.

[70] Griffioen K. J., Wan R., Okun E. (2011). GLP-1 receptor stimulation depresses heart rate variability and inhibits neurotransmission to cardiac vagal neurons. *Cardiovascular Research*.

[71] Liu L., Liu J., Tian X. Y. (2014). Uncoupling protein-2 mediates DPP-4 inhibitor-induced restoration of endothelial function in hypertension through reducing oxidative stress. *Antioxidants & redox signaling*.

[72] Yaribeygi H., Panahi Y., Javadi B., Sahebkar A. (2018). The underlying role of oxidative stress in neurodegeneration: a mechanistic review. *CNS & Neurological Disorders-Drug Targets (Formerly Current Drug Targets-CNS & Neurological Disorders).*.

[73] Yaribeygi H., Atkin S. L., Sahebkar A. (2019). A review of the molecular mechanisms of hyperglycemia-induced free radical generation leading to oxidative stress. *Journal of cellular physiology*.

[74] Hendarto H., Inoguchi T., Maeda Y. (2012). GLP-1 analog liraglutide protects against oxidative stress and albuminuria in streptozotocin-induced diabetic rats via protein kinase A-mediated inhibition of renal NAD (P) H oxidases. *Metabolism*.

[75] Liu J., Yin F., Zheng X., Jing J., Hu Y. (2007). Geniposide, a novel agonist for GLP-1 receptor, prevents PC12 cells from oxidative damage via MAP kinase pathway. *Neurochemistry International*.

[76] Shiraki A., Oyama J. I., Komoda H. (2012). The glucagon-like peptide 1 analog liraglutide reduces TNF-*α*-induced oxidative stress and inflammation in endothelial cells. *Atherosclerosis*.

[77] Koren S., Shemesh-Bar L., Tirosh A. (2012). The effect of sitagliptin versus glibenclamide on arterial stiffness, blood pressure, lipids, and inflammation in type 2 diabetes mellitus patients. *Diabetes technology & therapeutics*.

[78] Alam M. A., Chowdhury M. R. H., Jain P., Sagor M. A. T., Reza H. M. (2015). DPP-4 inhibitor sitagliptin prevents inflammation and oxidative stress of heart and kidney in two kidney and one clip (2K1C) rats. *Diabetology & metabolic syndrome*.

[79] Bian K., Doursout M. F., Murad F. (2008). Vascular system: role of nitric oxide in cardiovascular diseases. *The journal of clinical hypertension*.

[80] Ding L., Zhang J. (2012). Glucagon-like peptide-1 activates endothelial nitric oxide synthase in human umbilical vein endothelial cells. *Acta Pharmacologica Sinica*.

[81] Chai W., Dong Z., Wang N. (2012). Glucagon-like peptide 1 recruits microvasculature and increases glucose use in muscle via a nitric oxide–dependent mechanism. *Diabetes*.

[82] Dong Z., Chai W., Wang W. (2013). Protein kinase A mediates glucagon-like peptide 1-induced nitric oxide production and muscle microvascular recruitment. *American Journal of Physiology-Endocrinology and Metabolism*.

[83] Mason R. P., Jacob R. F., Kubant R., Ciszewski A., Corbalan J. J., Malinski T. (2012). Dipeptidyl peptidase-4 inhibition with saxagliptin enhanced nitric oxide release and reduced blood pressure and sICAM-1 levels in hypertensive rats. *Journal of cardiovascular pharmacology*.

[84] al-awar A., Almási N., Szabó R. (2018). Novel potentials of the DPP-4 inhibitor sitagliptin against ischemia-reperfusion (I/R) injury in rat ex-vivo heart model. *International journal of molecular sciences*.

[85] Tudurí E., Nogueiras R. (2017). Insulinotropic actions of GLP-1: how much in the brain and how much in the periphery?. *Endocrinology*.

[86] Göke R., Larsen P. J., Mikkelsen J. D., Sheikh S. P. (1995). Distribution of GLP-1 binding sites in the rat brain: evidence that exendin-4 is a ligand of brain GLP-1 binding sites. *European Journal of Neuroscience*.

[87] andrews C. N., bharucha A. E., camilleri M. (2007). Effects of glucagon-like peptide-1 and sympathetic stimulation on gastric accommodation in humans. *Neurogastroenterology & Motility*.

[88] Marney A., Kunchakarra S., Byrne L., Brown N. J. (2010). Interactive hemodynamic effects of dipeptidyl peptidase-IV inhibition and angiotensin-converting enzyme inhibition in humans. *Hypertension*.

[89] Palmer B. F. (2002). Renal dysfunction complicating the treatment of hypertension. *New England Journal of Medicine*.

[90] Muskiet M. H., Tonneijck L., Smits M. M. (2017). GLP-1 and the kidney: from physiology to pharmacology and outcomes in diabetes. *Nature Reviews Nephrology*.

[91] Boye K. S., Botros F. T., Haupt A., Woodward B., Lage M. J. (2018). Glucagon-like peptide-1 receptor agonist use and renal impairment: a retrospective analysis of an electronic health records database in the U.S. population. *Diabetes Therapy*.

[92] Dieter B. P., Alicic R. Z., Tuttle K. R. (2018). GLP-1 receptor agonists in diabetic kidney disease: from the patient-side to the bench-side. *American journal of physiology Renal, fluid and electrolyte physiology*.

[93] Hirata K., Kume S., Araki S. I. (2009). Exendin-4 has an anti-hypertensive effect in salt-sensitive mice model. *Biochemical and biophysical research communications*.

[94] Liu Q., Adams L., Broyde A., Fernandez R., Baron A. D., Parkes D. G. (2010). The exenatide analogue AC3174 attenuates hypertension, insulin resistance, and renal dysfunction in Dahl salt-sensitive rats. *Cardiovascular diabetology*.

[95] Said A., Hussain N., Ibrahim al Haddad A. H., Javid F. (2018). Effect of alogliptin on hypertensive chronic kidney disease patients with type 2 diabetes mellitus. *Australasian Medical Journal (Online)*.

[96] Alter M. L., Ott I. M., von Websky K. (2012). DPP-4 inhibition on top of angiotensin receptor blockade offers a new therapeutic approach for diabetic nephropathy. *Kidney and Blood Pressure Research*.

[97] Lee M.-Y., Tsai K.-B., Hsu J.-H., Shin S.-J., Wu J.-R., Yeh J.-L. (2016). Liraglutide prevents and reverses monocrotaline-induced pulmonary arterial hypertension by suppressing ET-1 and enhancing eNOS/sGC/PKG pathways. *Scientific Reports*.

[98] Woo J. S., Kim W., Ha S. J. (2013). Cardioprotective effects of exenatide in patients with ST-segment–elevation myocardial infarction undergoing primary percutaneous coronary intervention. *Arteriosclerosis, thrombosis, and vascular biology*.

[99] Pirozzi F., Dias M. (2015). Pulmonary artery relaxation was best with increasing GLP1 than the metabolic improvement in patients with type 2 diabetes. *Journal of Diabetes & Metabolism*.

[100] Honda J., Kimura T., Sakai S. (2017). The glucagon-like peptide-1 receptor agonist inhibits hypoxia-induced pulmonary hypertension in mice. *Journal of Cardiac Failure*.

[101] Hosokawa S., Haraguchi G., Maejima Y., Doi S., Isobe M. (2014). The synergistic effects of incretin-related drugs for the treatment of pulmonary arterial hypertension. *American Heart Association*.

[102] Skov J., Dejgaard A., Frøkiær J. (2013). Glucagon-like peptide-1 (GLP-1): effect on kidney hemodynamics and renin-angiotensin-aldosterone system in healthy men. *The Journal of Clinical Endocrinology & Metabolism*.

[103] Le Y., Zheng Z., Xue J., Cheng M., Guan M., Xue Y. (2016). Effects of exendin-4 on the intrarenal renin-angiotensin system and interstitial fibrosis in unilateral ureteral obstruction mice: exendin-4 and unilateral ureteral obstruction. *Journal of the Renin-Angiotensin-Aldosterone System*.

[104] Hubers S. A., Wilson J. R., Yu C. (2018). DPP (dipeptidyl peptidase)-4 inhibition potentiates the vasoconstrictor response to NPY (neuropeptide Y) in humans during renin-angiotensin-aldosterone system inhibition. *Hypertension*.

[105] Cooper M. E., Perkovic V., Groop P.-H. (2019). Hemodynamic effects of the dipeptidyl peptidase-4 inhibitor linagliptin with renin-angiotensin system inhibitors in type 2 diabetic patients with albuminuria. *Journal of hypertension*.

[106] Kim M., Platt M. J., Shibasaki T. (2013). GLP-1 receptor activation and Epac2 link atrial natriuretic peptide secretion to control of blood pressure. *Nature medicine*.

[107] Lovshin J. A., Barnie A., DeAlmeida A., Logan A., Zinman B., Drucker D. J. (2015). Liraglutide promotes natriuresis but does not increase circulating levels of atrial natriuretic peptide in hypertensive subjects with type 2 diabetes. *Diabetes Care*.

[108] Farr S., Taher J., Adeli K. (2014). Glucagon-like peptide-1 as a key regulator of lipid and lipoprotein metabolism in fasting and postprandial states. *Cardiovascular & Haematological Disorders-Drug Targets (Formerly Current Drug Targets-Cardiovascular & Hematological Disorders)*.

[109] Garber A., Henry R., Ratner R. (2009). Liraglutide versus glimepiride monotherapy for type 2 diabetes (LEAD-3 mono): a randomised, 52-week, phase III, double-blind, parallel-treatment trial. *The Lancet*.

[110] on behalf of the Liraglutide Effect and Action in Diabetes 5 (LEAD-5) met+SU Study Group, Russell-Jones D., Vaag A. (2009). Liraglutide vs insulin glargine and placebo in combination with metformin and sulfonylurea therapy in type 2 diabetes mellitus (LEAD-5 met+ SU): a randomised controlled trial. *Diabetologia*.

[111] Yang W., Chen L., Ji Q. (2011). Liraglutide provides similar glycaemic control as glimepiride (both in combination with metformin) and reduces body weight and systolic blood pressure in Asian population with type 2 diabetes from China, South Korea and India: a 16-week, randomized, doubl. *Diabetes, Obesity and Metabolism*.

[112] Marso S. P., Daniels G. H., Brown-Frandsen K. (2016). Liraglutide and cardiovascular outcomes in type 2 diabetes. *New England Journal of Medicine*.

[113] Hernandez A. F., Green J. B., Janmohamed S. (2018). Albiglutide and cardiovascular outcomes in patients with type 2 diabetes and cardiovascular disease (harmony outcomes): a double-blind, randomised placebo-controlled trial. *The Lancet*.

[114] Pfeffer M. A., Claggett B., Diaz R. (2015). Lixisenatide in patients with type 2 diabetes and acute coronary syndrome. *New England Journal of Medicine*.

[115] Holman R. R., Bethel M. A., Mentz R. J. (2017). Effects of once-weekly exenatide on cardiovascular outcomes in type 2 diabetes. *New England Journal of Medicine*.

[116] Marso S. P., Bain S. C., Consoli A. (2016). Semaglutide and cardiovascular outcomes in patients with type 2 diabetes. *New England Journal of Medicine*.

